# Coexistence of Two Distinct Tumor Types Within One Posterior Fossa Mass Lesion in an Adult Patient Verified by DNA-Methylation Analysis: A Case Report

**DOI:** 10.7759/cureus.35111

**Published:** 2023-02-17

**Authors:** Arwin Rezai, Johannes P. Pöppe, Mathias Spendel, Theo F. J. Kraus, Vlado Stevanovic, Christoph J. Griessenauer, Christoph Schwartz

**Affiliations:** 1 Department of Neurological Surgery, University Hospital Salzburg, Salzburg, AUT; 2 Department of Pathology, University Hospital Salzburg, Salzburg, AUT; 3 Department of Neuroradiology, University Hospital Salzburg, Salzburg, AUT

**Keywords:** subependymoma, case report, hemangioblastoma, dna-methylation-based classification, coexistence

## Abstract

We report an 81-year-old patient who underwent microsurgical resection of a posterior fossa mass lesion. Intraoperative findings were suggestive of the presence of two distinctly different tumor types within the lesion, one of which was well-circumscribed and avascular, whereas the other one showed an adhesive growth pattern and extensive vascularisation. Histopathological analysis, including deoxyribonucleic acid (DNA)-methylation-based classification, substantiated the intraoperative impression and confirmed the presence of a subependymoma central nervous system (CNS) World Health Organization (WHO) grade 1 as well as the presence of a hemangioblastoma CNS WHO grade 1. To our knowledge, our patient represents only the second reported case of such a rare constellation. Even though DNA-methylation-based classification is not yet required for the classification of all CNS tumor types by the 2021 WHO classification of tumors of the CNS, it proved to be crucial to verify the final diagnosis in our patient. In the future, DNA-methylation analysis will most likely become an important asset in neuro-oncological diagnostics and further help to guide treatment strategies in complex or rare clinical cases.

## Introduction

Posterior fossa tumors comprise approximately 15%-20% of all intracranial tumors in adults, with hemangioblastoma being the most common primary tumor type [1]. Hemangioblastoma is a tumor with neoplastic stroma cells and an abundance of small vessels [2]. They usually occur in the brain stem, cerebellum, and spinal cord and show a slow growth rate [2]. A much rarer tumor type (i.e. annual incidence 0.055 per 100,000) is the subependymoma, which may also occur in the posterior fossa/fourth ventricle [2-6]. Histopathologically, subependymomas are slow-growing, intraventricular glial tumors [2]. Both tumor types correspond to a WHO CNS grade 1 and are associated with a good prognosis if complete resection is achieved [2].

Even though DNA-methylation-based classification of neurooncological neoplasms is not required for all tumor types by the 2021 World Health Organization (WHO) classification of tumors of the central nervous system (CNS), it has become an increasingly important tool in neuro-oncological diagnostics and consequent guidance on patient treatment [2,7].

Here, we report a male adult patient who underwent neurosurgical resection of a posterior fossa mass lesion that intraoperatively displayed two surgically very distinct characteristics. Subsequent histopathological work-up, including DNA-methylation analyses, revealed a subependymoma as well as a hemangioblastoma, both corresponding to a WHO CNS grade 1, hereby confirming this very rare constellation of coexistence of two tumor entities within the same mass lesion. We provide detailed data on the performed surgery as well as the neuroradiological and neuropathological results. Written consent for publication of the case report was obtained.

## Case presentation

**Figure 1:**
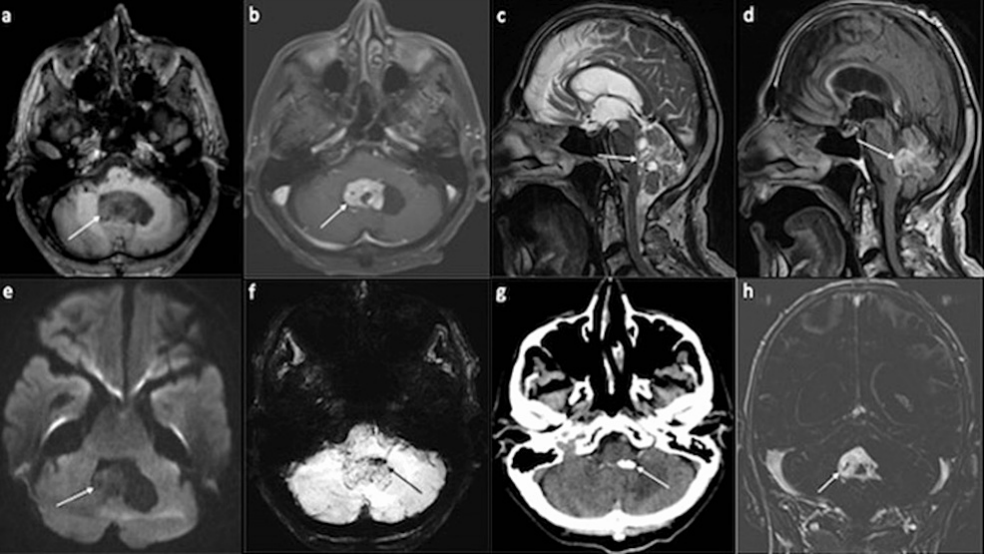
Preoperative magnetic resonance imaging and computed tomography Preoperative T1-weighted±contrast medium MRI revealed a T1-hypointense (Figure 1a) tumor with contrast-enhancement (Figure 1b) located in the fourth ventricle. On T2-weighted (Figure 1c) and fluid-attenuated inversion recovery (FLAIR) (Figure 1d) sequences, the tumor appeared hyperintense. A signal drop in apparent diffusion coefficient (ADC) mapping without restriction in diffusion-weighted imaging (DWI) was also noted (Figure 1e). Susceptibility-weighted imaging (SWI) was indicative of intratumoral calcifications (Figure 1f), which were confirmed by cCT (Figure 1g). T1-weighted MRI with fat-subtracted sequences showed no presence of significant intratumoral fat contents (Figure 1h).

An 81-year-old male patient was admitted to the trauma unit of a local hospital after suffering a conservatively treated pelvic fracture due to a fall. Because of a self-reported involuntary weight loss of 15 kg and declining general health within the last two months, cranial computed tomography (cCT) was performed, revealing a mass lesion in the fourth ventricle. A subsequent magnetic resonance imaging (MRI) showed a contrast-enhancing, partly cystic tumor (Figure 1). The patient was transferred to a superregional university hospital for neurosurgical treatment. Upon admission, the patient showed discreet dysarthria without other focal-neurologic deficits (i.e., Karnofsky Performance Score of 90). After receiving the patient’s written informed consent, he was scheduled to undergo tumor resection. A median suboccipital craniotomy followed by a telovelar approach to the fourth ventricle was performed. An avascular and well-circumscribed, pink-colored tumor was then exposed in the caudal portion of the fourth ventricle and carefully resected. A more cranial part of the tumor, however, was extensively vascularized and adherent to the medulla oblongata and the cerebellar peduncles. During the resection of this portion of the tumor, the patient became hemodynamically unstable (i.e., hypotension with bradycardia and asystole), and further tumor resection had to be aborted. The patient was transferred to the neurosurgical intensive care unit. After the patient’s condition had stabilized over the following days, a re-craniotomy was performed, and the residual tumor mass was successfully resected without further hemodynamic complications. Postoperatively, the patient required a tracheostomy due to the need for prolonged mechanical ventilation. Eventually, the patient’s condition slowly improved, the tracheostomy was closed, and he was transferred for further neurologic rehabilitation. 

Radiological analysis 

Based on the preoperative MRI and CCT, the most likely neuroradiological differential diagnoses were metastasis, ependymoma, and hemangioblastoma (Figure 1). A medulloblastoma and choroid plexus papilloma could have also been reasonable differential diagnoses but were deemed extremely unlikely due to the patient's age. The coexistence of two tumor types within the lesion was not suspected based on the available preoperative MRI data.

Surgery

As described above, two surgeries were necessary to achieve gross total resection of the tumor. During the first surgery, we achieved good exposure to the caudal part of the lesion, and the resection went smoothly. Initially, the tumor was easily dissected from the brainstem and the floor of the fourth ventricle. When reaching the cranial part, however, the tumor became increasingly vascularized and adherent to the surrounding structures. For the second surgery, an external ventricular drain was placed as a security measure in case of intraventricular bleeding, and intraoperative neuromonitoring (IOM) was used to minimize brainstem manipulation. In hindsight, IOM should have been already used for the initial surgery to reduce the risk of surgery-associated neurologic deterioration.

Histopathology and molecular genetic analysis

**Figure 2:**
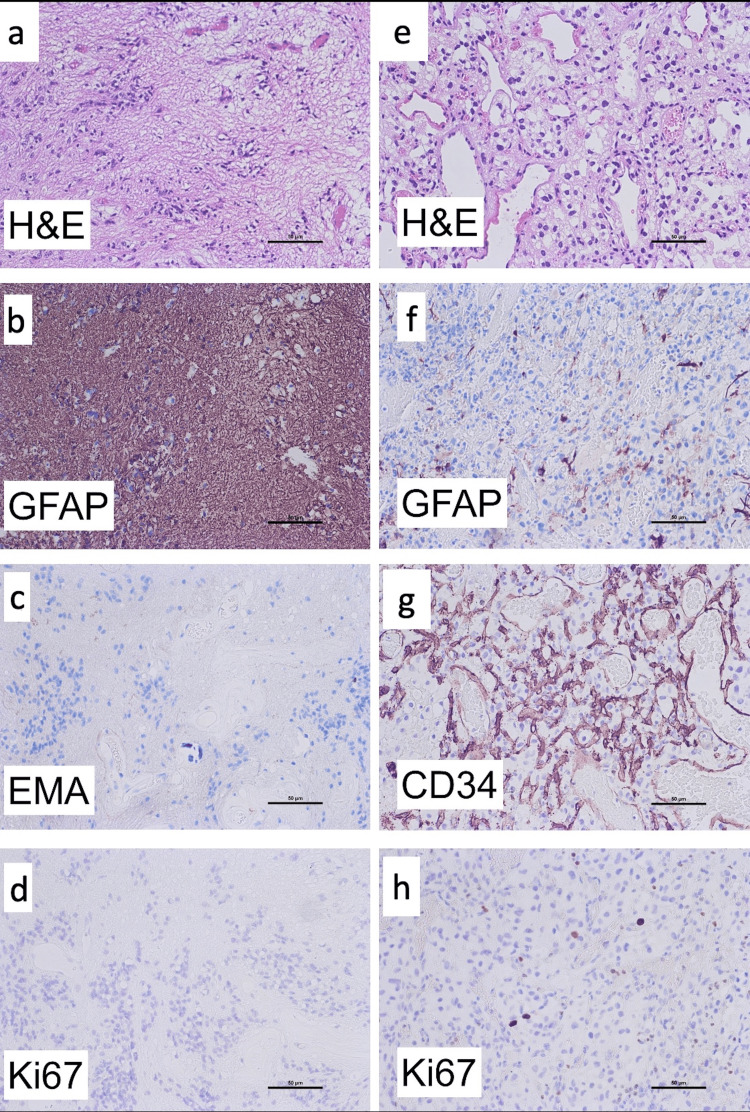
Histopathology and molecular genetic analysis Figures a-d: Neuropathological analysis of the tissue obtained in the first surgery showed a glial tumor with clusters of small monomorphic nuclei without any necrosis (Figure 2a). Expression of GFAP proved the tissue to be from the glial origin (Figure 2b). The very rare expression of EMA was seen, which would well suit to a tumor of benign origin (Figure 2c), and also a low proliferation was seen (Figure 2d). Figures e-h: Neuropathological analysis of the tissue obtained in the second surgery showed a well-vascularized tissue and areas with hemorrhage (Figure 2e). The tissue showed to have only a partial expression of GFAP (Figure 2f) contrary to the tissue obtained in the first surgery. Antibodies against CD34 showed a rich vascularized tissue (Figure 2g), and a low proliferation was seen (Figure 2h). H&E: hematoxylin and eosin; GFAP: glial fibrillary acidic protein; EMA: epithelial membrane antigen

**Figure 3:**
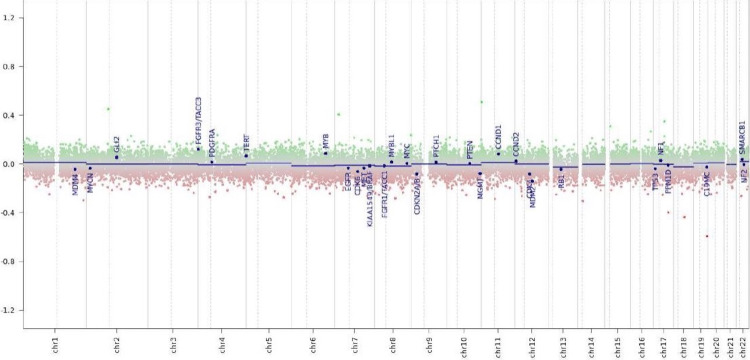
Molecular genetic analysis using DNA-methylation profiling showed no chromosomal gains or losses in copy number variation analysis consistent with the diagnosis of a subependymoma x-axis: chromosomes y-axis: log2 copy number ratio

Neuropathological analysis of the tissue obtained in the first surgery showed a glial tumor with clusters of small, monomorphic nuclei that were embedded in a loose fibrillary matrix in hematoxylin and eosin (H&E) stains (Figure 2a). No mitosis or necrosis was seen. Immunohistochemistry proved glial origin with an expression of the glial fibrillary acidic protein (GFAP) (Figure 2b), and there was a very rare expression of epithelial membrane antigen (EMA) (Figure 2c). Proliferation activity was low, with less than 1% of tumor cells expressing Ki67 (Figure 2d). Molecular genetic analysis using DNA-methylation profiling showed no chromosomal gains or losses in copy number variation (CNV) analysis and allocated the tumor to the molecular class of subependymoma with a calibrated score of 1.00 (Figure 3 and Table 1).

**Table 1:** Methylation classifier result (v11b4)

Methylation Class	Calibrated Score	Interpretation
Subependymoma, posterior fossa	1.00	Match
Hemangioblastoma	0.94	Match

**Figure 4:**
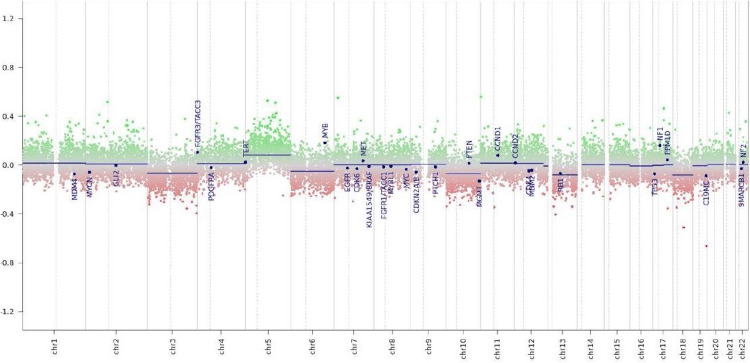
Molecular genetic analysis using DNA-methylation profiling showed some minor chromosomal gains and losses in copy number variation analysis in accordance with a hemangioblastoma x-axis: chromosomes y-axis: log2 copy number ratio

Neuropathological analysis of the tissue obtained in the second surgery showed large areas with hemorrhage and well-vascularized tissue in H&E stains (Figure 2e). Furthermore, there were regions with cohesive cell aggregates with blurred eosinophilic cytoplasm. Immunohistochemistry showed only partial expression of GFAP (Figure 2f). Antibodies against CD34 showed a rich vascularized tissue (Figure 2g), and proliferation using Ki67 was low (Figure 2h). Molecular genetic analysis using DNA-methylation profiling showed some minor chromosomal gains and losses in CNV analysis and allocated the tumor to the group of hemangioblastoma with a calibrated score of 0.94 (Figure 4 and Table 1).

Thus, histopathological examinations revealed two distinct tumor types within the resected mass lesion, a subependymoma CNS WHO grade 1 and a hemangioblastoma CNS WHO grade 1. Due to the histopathological results and postoperative MRI confirming gross total resection, no further adjuvant treatment was deemed necessary.

## Discussion

We believe this case provides several interesting aspects of surgery and intraoperative findings, as well as neuroradiological and neuropathological diagnostics. A preoperative cCT and cranial MRI scan showed an intraventricular mass lesion in the posterior fossa. Based only on the preoperative imaging, a definitive suspected diagnosis could not be obtained. Due to the intraoperative ambiguous findings, with one tumor being avascular and well-circumscribed while the other one being extensively vascularized and adherent, the possibility of two completely different tumor types was already discussed between the operating surgeons. This presumption was eventually confirmed by neuropathological examination via DNA-methylation profiling. 

Our case represents the second reported patient with such a rare histological constellation with the coexistence of a subependymoma and a hemangioblastoma within one lesion. To the best of our knowledge, only one similar case has been reported thus far, dating back to 1985, and the incidence of such histopathological constellations remains unclear [8]. Furthermore, our results with regard to the histopathological diagnosis could be substantiated by the application of a recently published tool, the DNA-methylation-based classification [7,9]. DNA-methylation analyses have been increasingly introduced in neuro-oncology over the last five years [7,9]. For instance, the DNA methylation-based analysis of meningiomas, grading them into subgroups, showed to be more accurate in the prediction of prognosis and recurrent disease compared to the WHO grading [10]. In our case, the use of DNA methylation-based analysis significantly helped to determine the final histopathological diagnoses. This aspect was crucial for further decision-making with regard to follow-up and/or potential adjuvant therapy, which was not deemed necessary in our case. The prognosis and further treatment decisions are determined by both histopathologies in these rare constellations; generally speaking, the more malignant tumor will most likely determine the patient's outcome. In our patient, due to the finding of a hemangioblastoma, a further work-up (i.e., ophthalmoscopy, abdominal ultrasound, and genetic testing) to screen for Von Hippel-Lindau syndrome was also scheduled but had not been performed before transfer to neurological rehabilitation.

Although DNA methylation-based analysis is not being implemented in the WHO grading of all CNS tumors [7,9], we believe that in the future, this tool will further gain importance to obtain the best possible treatment of patients with CNS tumors. 

During the initial tumor resection, we encountered severe difficulties due to the highly vascularized nature of the lesion and the patient's cardiovascular instability. The hemodynamic instability was most likely caused by manipulations of the area of the fourth ventricle during the attempted tumor resection leading to recurrent bradycardia, hypotension, and once a brief episode of asystole. After consulting with the anesthesiologist, the surgery was terminated, and a second surgery was planned after the stabilization of the patient the next days. In hindsight, IOM, as used in the second surgery, may have been helpful in the earlier detection of these complications.

## Conclusions

The coexistence of two distinct tumor types within one lesion is a very rare but possible phenomenon. This may pose special challenges to neurosurgeons if not identified in preoperative imaging. In this complex constellation, DNA-methylation profiling may be crucial to determine the eventual histopathological diagnosis and substantiate intraoperative findings allowing for optimal patient treatment.
